# Notochord Injury Assays that Stimulate Transcriptional Responses in Zebrafish Larvae

**DOI:** 10.21769/BioProtoc.3100

**Published:** 2018-12-05

**Authors:** Zhiqiang Zeng, Juan C Lopez-Baez, Laura Lleras-Forero, Hannah Brunsdon, Cameron Wyatt, Witold Rybski, Nicholas D Hastie, Stefan Schulte-Merker, E Elizabeth Patton

**Affiliations:** 1 MRC Human Genetics Unit, MRC Institute of Genetics and Molecular Medicine, University of Edinburgh, Crewe Road, Edinburgh, EH4 2XU, The United Kingdom; 2 CRUK Edinburgh Centre, MRC Institute of Genetics and Molecular Medicine, University of Edinburgh, Crewe Road, Edinburgh, EH4 2XU, The United Kingdom; 3 Hubrecht Institute - KNAW & UMC Utrecht, Utrecht, Netherlands; 4 Faculty of Medicine, Institute for Cardiovascular Organogenesis and Regeneration, WWU Münster, Münster, Germany; 5 CiM Cluster of Excellence, Münster, Germany

**Keywords:** Zebrafish, Notochord, Tail fin, Injury, Amputation, Tissue repair, Tungsten wire, Nystatin

## Abstract

Zebrafish have become an increasingly important model organism in the field of wound healing and regenerative medicine, due to their high regenerative capacity coupled with high-resolution imaging in living animals. In a recent study, we described multiple physical and chemical methods to induce notochord injury that led to highly specific transcriptional responses in notochord cellular subpopulations. The notochord is a critical embryonic structure that functions to shape and pattern the vertebrae and spinal column. Here, we describe precision needle injury, tail-notochord amputation, and chemical inhibition of caveolin that trigger a wound-specific *wt1b* expression response in the notochord sheath cell subpopulation. We propose that these procedures can be used to study distinct cell populations that make up the cellular processes of notochord repair.

## Background

**Figure 1:**
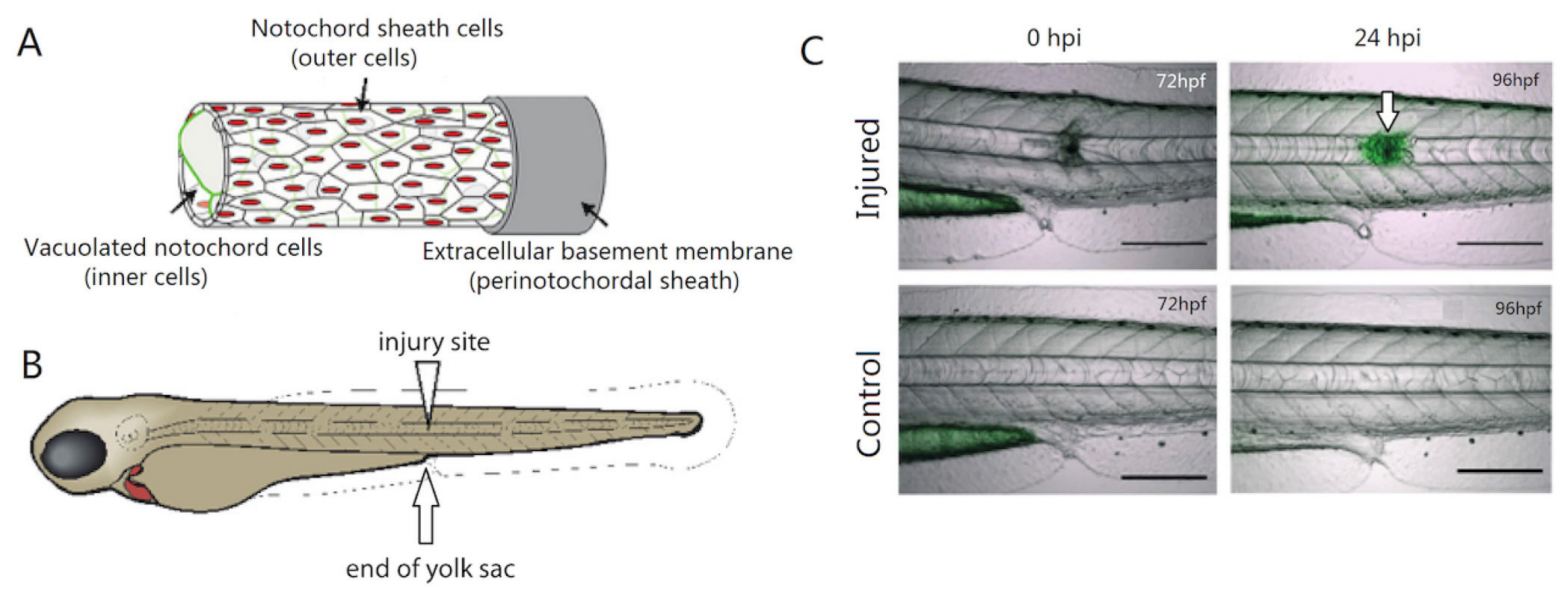
Cell populations of the notochord and the *wt1b* notochord wound response. A. Schematic of the cell populations of the notochord. The notochord is comprised of two physically distinct cell populations: an epithelial-like notochord sheath cell population (outer cells; red) and a large vacuolated notochord cell population (inner cells, green), which are tightly wrapped by a thick, elastic extracellular basement membrane (peri-notochordal sheath). B. Schematic of the zebrafish embryo and the site of the notochord wound at the end of the yolk sac (YS). C. Needle injury triggers localized *wt1b:gfp* expression in the notochord at the site of damage by 24 h post injury (hpi; arrow). Scale bars = 100 µm in Panel C.

The notochord is a transient embryonic structure that provides axial support and signaling information to the developing embryo (Ellis *et al.*, 2013). It is comprised of two structurally distinct cell populations: the inner vacuolated cells that provide embryo support and structure, and the outer sheath cells that maintain turgor pressure for the vacuolated cells as well as patterning the developing vertebrate spine (Wopat *et al.*, 2018; Lleras Forero *et al.*, 2018; Figure 1A). We have recently discovered that *wilms' tumor 1b* (*wt1b*) is specifically expressed in a notochord sheath cell subpopulation that emerges at the site of damage and is maintained throughout repair and formation of adult vertebra structure in zebrafish (Figures 1B and 1C; Lopez-Baez *et al.*, 2018). WT1 is a zinc-finger transcription factor involved in mesodermal tissue development, adult tissue homeostasis, and becomes reactivated during epicardial tissue damage (Hastie *et al.*, 2017). Our discovery that *wt1b* becomes expressed at the notochord wound may have important implications for the development of therapies for vertebrae spinal injuries or degenerative processes.

In zebrafish wounding and regeneration models, injury is induced by a variety of methods such as amputation, surgical resection, irradiation, laser ablation and genetic ablation (Gemberling *et al.*, 2013). For example, in larval zebrafish, syringe needles of various sizes have been used for tail fin amputation and spinal cord injury experiments (Lisse *et al.*, 2015; Wehner *et al.*, 2017).

We have conducted notochord injury assays in zebrafish larvae using physical and chemical approaches (Lopez-Baez *et al.*, 2018). Electrolysis-sharpened tungsten wire and insect pins described here and in our recent paper induce precise, localized injury and trigger wound-specific *wt1b* expression (Figures 1B and 1C). The structural integrity of the notochord can also be disrupted chemically by treating embryos with nystatin, a small molecule which binds sterols and disassembles caveolae (Rothberg *et al.*, 1992), which are particularly abundant in the notochord (Lim *et al.*, 2017). We detected increased *wt1b* expression in nystatin treated notochords suggesting changes in caveolae caused by non-physical damage and stress may also induce *wt1b* expression.

## Materials and Reagents

1. Ø 0.25 mm Tungsten wire (Alfa Aesar, catalog number: 010073.G2)
2. Metal needle holder (VWR International Ltd. UK, catalog number: MURRL110/01)
3. Sterile scalpel blade (Swann Morton UK, catalog number: 11708353)
4. 0.10 mm Austerlitz insect pins, stainless steel (Fine Science Tools, catalog number: 26002-10)
5. Glass Pasteur Pipettes length: 145 mm (Brand, catalog number: 7477 15)
6. Petri dish (Thermo Scientific, catalog number: 15370366)
7. 96-well plate
8. Zebrafish larvae (3-7 days post fertilization)
  Non-pigmented *mitfa* mutant (*nacre* allele) (Lister *et al.*, 1999) may be preferable for ease of imaging. For our experiments, we used the Tg(R2*col2a1a:mCherry*) transgenic line to visualize notochord sheath cells (Dale and Topczewski, 2011), and the Tg(*wt1b:gfp*) line to study the wound response in the notochord (Perner *et al.*, 2007).
  *Important: Zebrafish older than 5 days post fertilization are protected animals by UK and EU law and require proper animal procedure licenses and approval from institutional ethics committees*.
9. Dimethylsulfoxide (DMSO) (Sigma-Aldrich, catalog number: D2650-100ML)
10. Nystatin (Sigma-Aldrich, catalog number: N6261-500KU)
11. Agarose (Invitrogen, catalog number: 15510-027)
12. Tricaine (MS-222, 3-amino benzoic acid ethyl ester, Sigma, catalog number: A-5040)
13. NaOH pellets (Sigma-Aldrich, catalog number: S8045-500G)
14. Sodium chloride (NaCl) (Sigma-Aldrich, catalog number: S7653-1KG)
15. Potassium chloride (KCl) (Sigma-Aldrich, catalog number: P9333-500G)
16. Calcium chloride dihydrate (CaCl_2_·2H_2_O) (Sigma-Aldrich, catalog number: 223506-500G)
17. Magnesium chloride hexahydrate (MgCl_2_·6H_2_O) (Sigma-Aldrich, catalog number: M2393-500G)
18. E3 embryo medium (60x stock solution) (see Recipes)
19. Tricaine (MS-222) (1x working solution) (see Recipes)
20. NaOH (5 M) (see Recipes)

## Equipment

**Figure 2:**
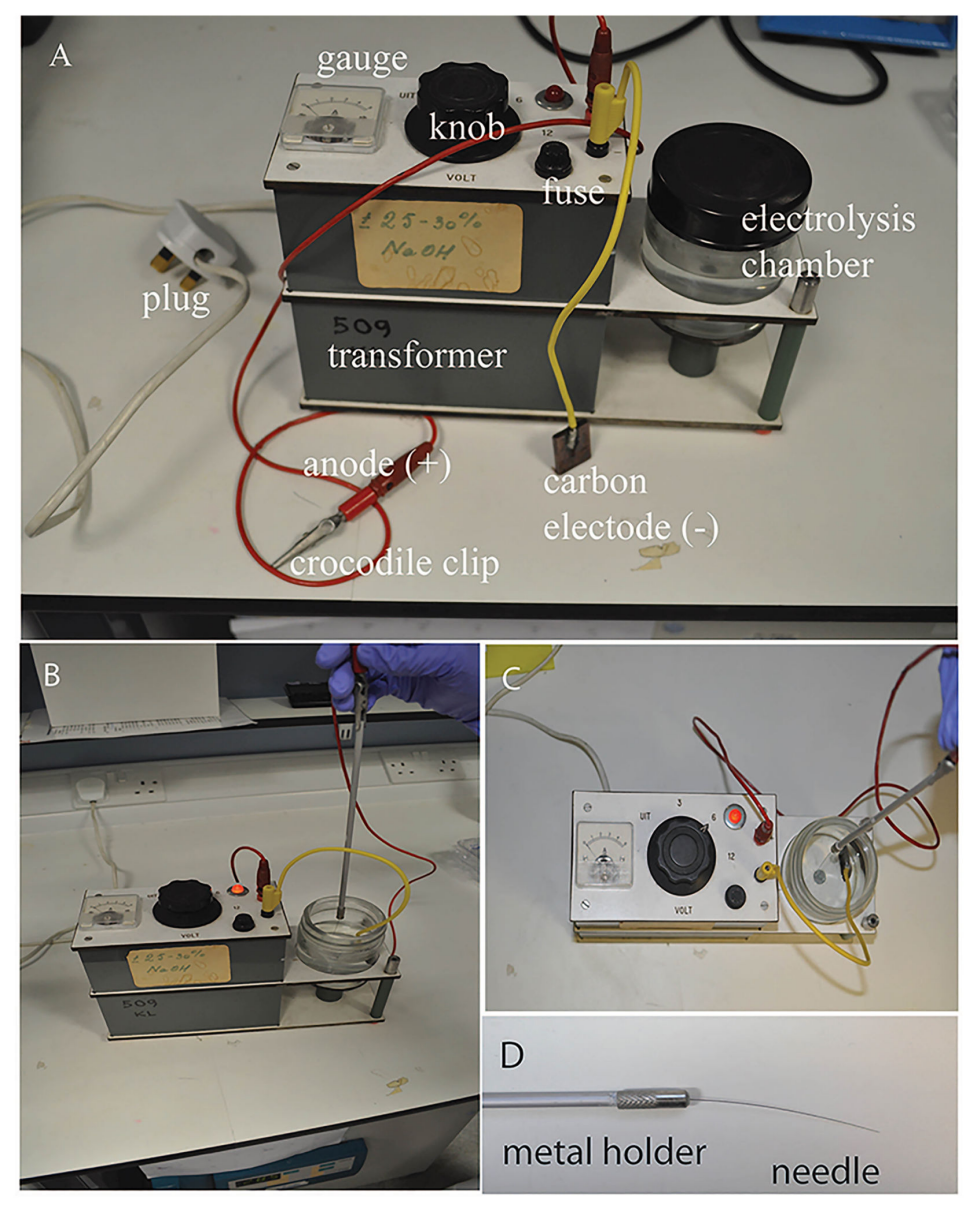
Preparation of electrolysis-sharpened needle. A. Electrolysis-based apparatus, consisting of a rectified DC transformer, an anode with crocodile clip (red), a cathode with carbon electrode (yellow), electrolysis chamber with 5 M NaOH electrolyte. B-C. Lateral and dorsal views of a tungsten wire needle being sharpened. With the power on (6 V), the mounted needle is held vertically and dipped into and out of the electrolyte steadily and slowly until the desired tip is achieved. D. A finished electrolysis-sharpened tungsten wire needle.

1. 250 ml glass bottle
2. Forceps
3. Microwave oven
4. Incubator
5. Bunsen burner
6. Microscopes:
  Upright Stereomicroscope (*e.g.*, Nikon, model: SM2645)
  Light microscope (*e.g.*, Olympus, model: SZX16)
  Confocal microscope (*e.g.*, Nikon, model: A1R)
7. Electrolysis device for tungsten wire sharpening (the device shown in this protocol is custom-made and no longer in production; Figure 2A)
  However, users can set up their own equipment.
  - Parts required are: a) DC power supply (3-20 V, such as Bosch C3 smart car battery charger, catalog number: 0092C35000), b) carbon electrode rod (Lasec, catalog number: ERDI9470), c) crocodile clips (optional, DC power supply may come with leads with crocodile clips), d) a 200 ml glass container (jar) with lid.
  - Assemble the equipment: Plug the leads into the charger. Using crocodile clips, connect the carbon electrode rod to the negative terminal (black lead), connect the metal holder to the positive terminal (red lead). Set the voltage to 6 V and fill up the jar with 100 ml of NaOH solution. The equipment is now ready for use.

## Procedure

**Figure 3:**
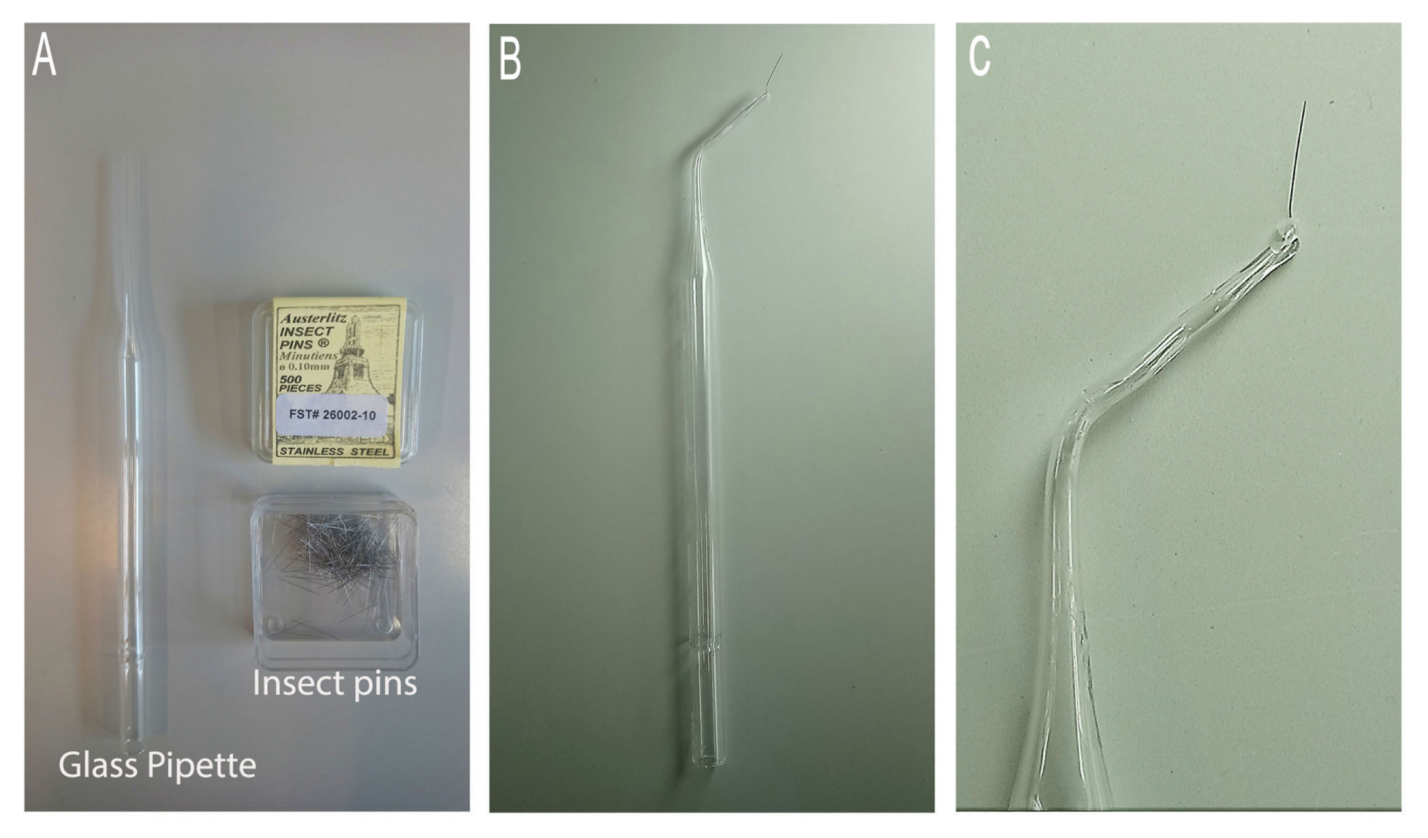
Preparation of insect pin. A. The required equipment. B. The finished instrument. C. A close up image of the tip.

**Figure 4:**
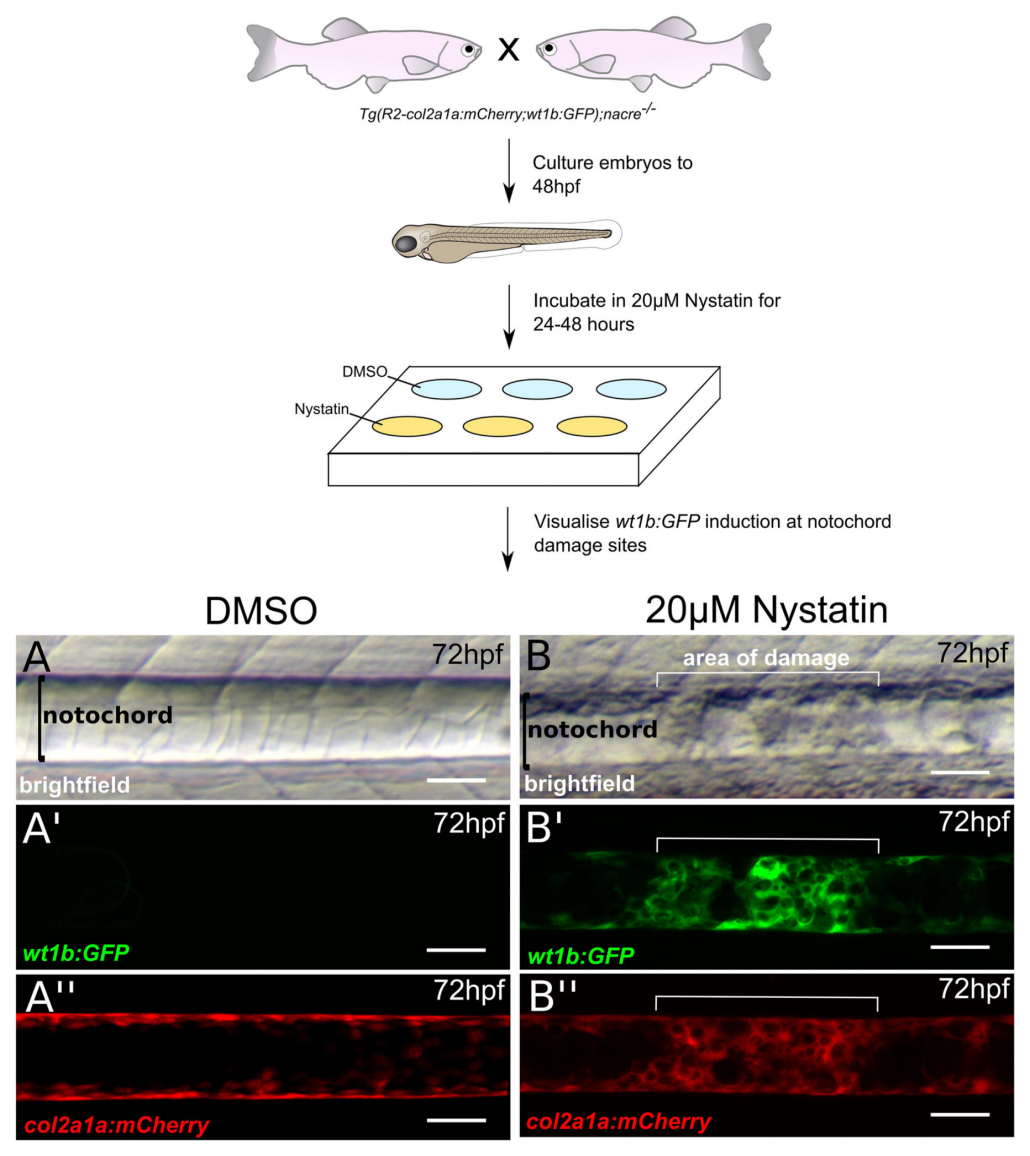
Disrupting notochord structure using nystatin (modified from Lopez-Baez, 2018). Nystatin is a small molecule which binds sterols and leads to the disassembly of caveolae, a component abundant in the notochord (Lim *et al.*, 2017). Tg(*wt1b:GFP;R2-col2a1a:mCherry);nacre^-/-^* zebrafish embryos are treated with either DMSO or 20 μM nystatin from 48 hpf to 72 hpf. When observed under a light microscope, the notochord structure of (A) DMSO-treated embryos appears normal, however lesions can be observed in (B) nystatin-treated embryos. (A’ and B’) *wt1b:GFP* expression is induced at lesion sites, but not in control notochords. *R2-col2a1a:mCherry* expression in notochord sheath cells also shows increased cellularity at (B”) lesion sites of nystatin-treated embryos compared to (A”) DMSO controls. Scale bars are 50 μm.

**Figure 5:**
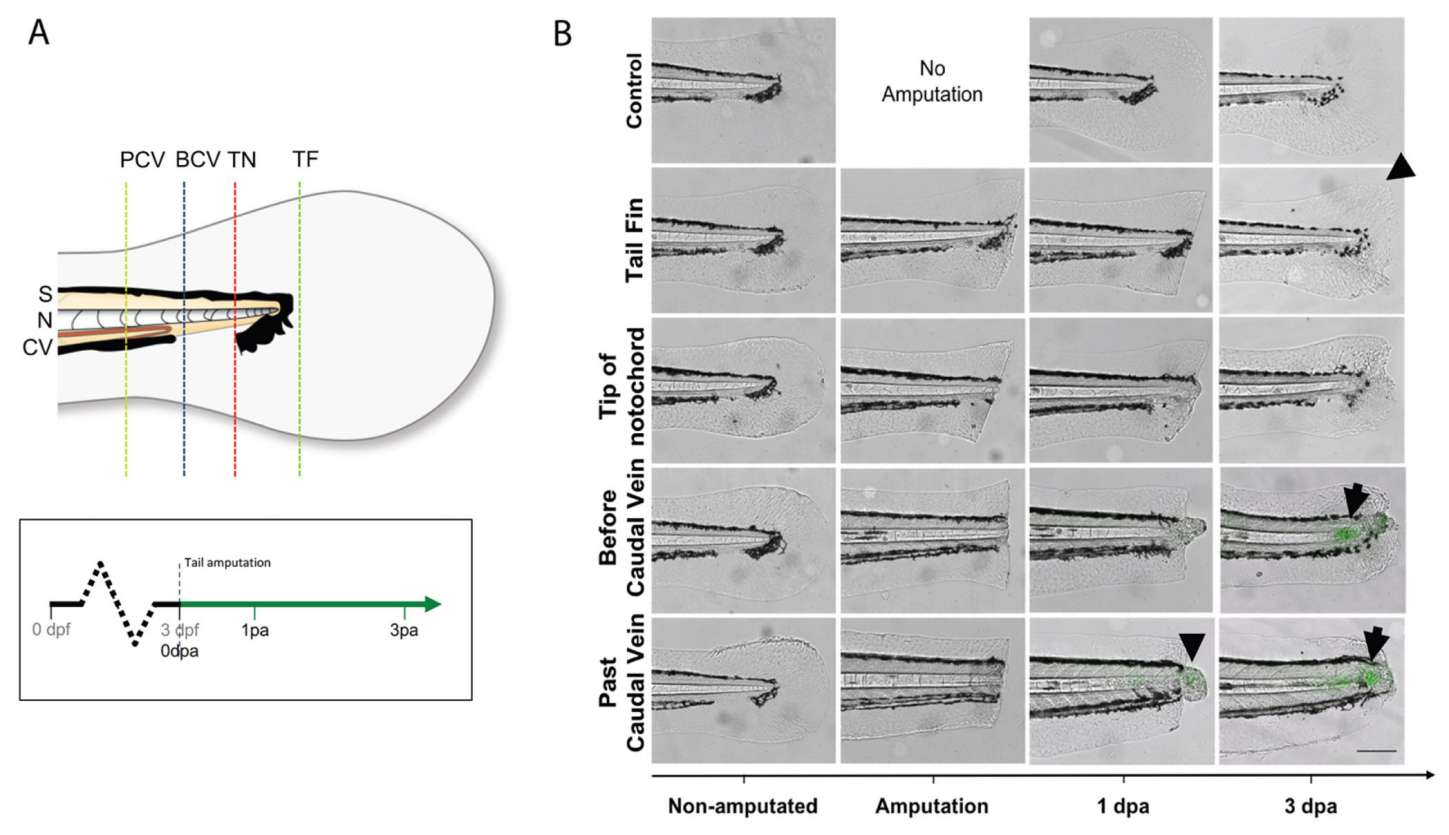
Selected tail amputations uncover the notochord specificity of the response (modified from Lopez-Baez, 2018). A. Illustration of tail amputations at different tail sites and time points when images are taken. Tail fin (TF), tip of the notochord (TN), before caudal vein (BCV), past caudal vein (PCV), somite (S), notochord (N), caudal vein (CV). B. TF and TN amputated larvae showed no GFP upregulation in their notochord after the injury, but show marked fin regeneration (arrow head). BCV and PCV amputated groups both showed strong GFP upregulations by 72 hpa (arrows), with PCV amputated larvae showing an overall stronger and faster upregulation than BCV amputated larvae.

- Notochord needle injury of zebrafish larvae
  1. Prepare electrolysis-sharpened tungsten wire (adapted from Brady, 1965) (Figure 2 and Video 1)
    - Cut off 3 cm of tungsten wire and mount it into a needle holder.
    - Connect the metal handle of the needle holder to the (+) terminal of the transformer using a crocodile clip, connect the cathode with carbon plate to the (-) terminal.
    - Place the carbon electrode in the glass chamber with 100 ml of 5 M NaOH solution and switch on the transformer, set the output voltage to 6 V. With the mounted needle held vertically, dip the needle into and out of NaOH, slowly and steadily until desired tip is produced. Faster movement = Longer slope on needle, Slower movement = Shorter tip with more angled slope. The dial gauge of current reads between 0 and 1 A when the needle moves up and down. It takes about 2.5 min to sharpen a needle from 0.25 mm to 0.02 mm in diameter.
  2. Alternatively, 0.1 mm insect pins can also be used to injure the notochord (Figure 3 and Video 2)
    - Take a clean glass pipette and using a Bunsen burner, bend the thin side in the middle in order to create a 45-degree angle. This will help with the injury manipulation procedure.
    - Close the hole of the thin side by about three-quarters using the Bunsen burner. This is done by placing the tip in the strongest part of the flame and rotating it in a circular manner.
    - Take the insect pin with forceps and place it in the hole, taking care that the sharp side is facing the outside.
    - Carefully continue burning the tip of the glass pipette in the weakest part of the flame, until the hole is closed and the insect pin secure. The insect pin will burn if exposed to too much heat. Try to close the hole as much as possible before inserting the pin.
  3. Prepare 1.5% agarose
    Weight 1.5 g agarose and melt in 100 ml E3 embryo medium in a 250 ml glass bottle using a microwave oven, pour a thin layer into a Ø 90 mm Petri dish. About 20 ml of 1.5% agarose is used. Let the agarose solidify.
  4. Anaesthetize larvae in tricaine solution
    Prepare 1:10,000 tricaine solution using E3 embryo medium (please see Recipes), and pour 30 ml in a separate Ø 90 mm Petri dish. Transfer one larva into the tricaine solution and wait until it is anesthetized.
  5. Under a stereomicroscope, place one larva on its side on a Petri dish coated with agarose so that the lateral side can be accessed with needle from above. Remove as much liquid as possible so that the surface tension adheres the larvae to the dish and prevents it from slipping. Gently insert the tip of the tungsten wire into the notochord vertically at the level of the end of the yolk sac (Figure 1B), then withdraw the wire. (Video 3)
  6. Transfer injured larvae to a Petri dish with fresh E3 medium to recover and place the dish at 28.5 °C to grow the larvae to the desired stages. Keep uninjured age-matched larvae as non-injured controls.
- Chemically-induced disruption of notochord
  1. Cross fish carrying the *Tg*(*wt1b:GFP*) and the notochord-marking *Tg*(*R2-col2a1a:mCherry*) transgenes in an unpigmented *nacre^-/-^* background, to obtain *Tg(wt1b:GFP;R2-col2a1a:mCherry);nacre^-/-^* embryos.
  2. Prepare fresh 5 mg/ml nystatin stock solution (5.4 mM) before each use by dissolving in DMSO.
  3. Dilute nystatin stock solution in E3 to obtain 20 μM final working concentration. Add this to dechorionated 48 hpf embryos in a 6-well plate. Add 0.4% DMSO to control embryos.
  4. Incubate embryos at 28.5 °C for up to 48 h. After 24 h of nystatin treatment, lesions appear along the length of the notochord. They tend to appear first in regions that are naturally compressed as the embryo moves, and then spread along the length of the notochord. The majority of embryos acquire notochord lesions, however their size and severity can be variable. Therefore, regular screening for lesions and/or the onset of *wt1b:GFP* expression is recommended in order to identify embryos with the desired level of notochord damage.
  5. For imaging, anesthetize embryos in tricaine (1:10,000), and mount sagittally in 1% low-melt agarose. Brightfield images are taken using a light microscope (Figures 4A and 4B). Expression of the *R2-col2a1a:mCherry* transgene, which marks the notochord, and the induction of the *wt1b:GFP* transgene at sites of notochord damage is visualized using confocal microscopy (Figures 4A’ and 4B”).
- Tail amputation
  1. Prepare 1.5% agarose using E3 embryo medium and pour a thin layer into a Petri dish. Let the agarose solidify.
  2. Anaesthetize larvae in tricaine solution.
  3. Under a stereomicroscope, place one larva on its side onto the solidified agarose. Remove as much as liquid as possible, so the surface tension adheres the larvae to the dish and prevents it from slipping, then amputate the tail with a sterile scalpel blade with slight pressure. Amputation sites are dependent on experiments being performed (Figure 5). Amputations at the tail fin and tip of notochord site do not include notochord tissue, and do not stimulate a *wt1b:gfp* notochord injury response. Amputations beyond the tail fin and into the notochord (before caudal vein, past caudal vein) stimulate a *wt1b:gfp* expression.
  4. Transfer injured larvae to a Petri dish with fresh E3 medium to recover, and place the dish at 28.5 °C to grow the larvae to the desired stages. Keep uninjured age-matched larvae as non-injured controls.

## Notes

1. In the UK and EU, all animal procedures need to be approved by the Home Office (UK) or its equivalent. Appropriate Personal Project License (PPL) and Personal individual License (PIL) are required.
2. The procedure of notochord needle injury requires a fair amount of practice, and care should be taken to not cause injury outside the needle injury site. The appearance of a small bulge structure at the site of injury within the notochord about 5 min post-surgery indicates a successful operation. It is achievable to injure 30 larvae during a period of an hour.
3. Optimizing nystatin dosage for the first-time use is recommended, as there is batch-to-batch variation. A longer nystatin incubation period can be attempted however the adverse off-target effects of nystatin cause gross developmental abnormalities and embryos do not survive long-term. In our hands, treatment with 20 μM nystatin from 48 hpf for 24 h gives the most consistent results. Treatment with nystatin before 48 hpf is possible, however, due to its off-target effects, more toxicity is seen. Embryos tolerate later nystatin treatment from 72 and 96 hpf much better, with 20 μM nystatin producing notochord lesions in 60%-80% of embryos after 24 h, although these lesions are smaller in size and fewer per embryo compared those shown in Figure 4.

## Recipes

1. E3 embryo medium (60x stock solution)
  17.4 g NaCl
  0.8 g KCl
  2.9 g CaCl_2_·2H_2_O
  4.89 g MgCl_2_·6H_2_O
  Dissolve in 1 L H_2_O
2. Tricaine (MS-222) (1x working solution)
  Dissolve 0.1 g of Tricaine powder in 1 L of 1x E3 medium, adjust pH to 7.0
3. NaOH (5 M)
  Dissolve 200 g NaOH pellets in 1 L H_2_O

## Supplementary Material

Video 1 Sharpening tungsten wire

Video 2 Preparing insect pin

Video 3 Notochord Needle Injury
